# Driving pressure, as opposed to tidal volume based on predicted body weight, is associated with mortality: results from a prospective cohort of COVID-19 acute respiratory distress syndrome patients

**DOI:** 10.62675/2965-2774.20240208-en

**Published:** 2024-04-22

**Authors:** Erich Vidal Carvalho, Maycon Moura Reboredo, Edimar Pedrosa Gomes, Pedro Nascimento Martins, Gabriel Paz Souza Mota, Giovani Bernardo Costa, Fernando Antonio Basile Colugnati, Bruno Valle Pinheiro

**Affiliations:** 1 Universidade Federal de Juiz de Fora Hospital Universitário Pulmonary and Critical Care Division Juiz de Fora MG Brazil Pulmonary and Critical Care Division, Hospital Universitário, Universidade Federal de Juiz de Fora - Juiz de Fora (MG), Brazil.

**Keywords:** Acute respiratory distress syndrome, COVID-19, Coronavirus infections, Tidal volume, Respiration, artificial, Mortality, Intensive care units

## Abstract

**Objective:**

To evaluate the association between driving pressure and tidal volume based on predicted body weight and mortality in a cohort of patients with acute respiratory distress syndrome caused by COVID-19.

**Methods:**

This was a prospective, observational study that included patients with acute respiratory distress syndrome due to COVID-19 admitted to two intensive care units. We performed multivariable analyses to determine whether driving pressure and tidal volume/kg predicted body weight on the first day of mechanical ventilation, as independent variables, are associated with hospital mortality.

**Results:**

We included 231 patients. The mean age was 64 (53 - 74) years, and the mean Simplified Acute and Physiology Score 3 score was 45 (39 - 54). The hospital mortality rate was 51.9%. Driving pressure was independently associated with hospital mortality (odds ratio 1.21, 95%CI 1.04 - 1.41 for each cm H_2_O increase in driving pressure, p = 0.01). Based on a double stratification analysis, we found that for the same level of tidal volume/kg predicted body weight, the risk of hospital death increased with increasing driving pressure. However, changes in tidal volume/kg predicted body weight were not associated with mortality when they did not lead to an increase in driving pressure.

**Conclusion:**

In patients with acute respiratory distress syndrome caused by COVID-19, exposure to higher driving pressure, as opposed to higher tidal volume/kg predicted body weight, is associated with greater mortality. These results suggest that driving pressure might be a primary target for lung-protective mechanical ventilation in these patients.

## INTRODUCTION

Patients with acute hypoxemic respiratory failure due to COVID-19 frequently fulfill the criteria for acute respiratory distress syndrome (ARDS) diagnosis, according to the Berlin definition, and are mechanically ventilated.^(1-3)^ Although essential for treating patients with ARDS of different etiologies, including COVID-19, mechanical ventilation (MV) can worsen inflammatory lung injury, a phenomenon known as ventilator-induced lung injury (VILI).^(4)^ Several studies conducted in ARDS patients have shown an association between different ventilatory parameters, such as tidal volume (V_T_), plateau pressure (P_plat_), driving pressure (DP), mechanical power (MP), and mortality.^(5-8)^ Based on these studies, ventilatory settings should aim not only to correct hypoxemia but also to protect the lungs from VILI.^(9)^

At the beginning of the pandemic, two different phenotypes were described among patients with COVID-19 who developed acute hypoxemic respiratory failure. While some patients had typical ARDS presentations, with high elastance, high lung weight and greater potential for alveolar recruitment, others, for the same level of hypoxemia, presented low elastance and low lung weight, with lower potential for alveolar recruitment.^(10,11)^ At that time, it was not clear whether ARDS patients with COVID-19 should be ventilated with the same principles described for ARDS patients with other etiologies. However, several other studies have shown that patients with COVID-19-related ARDS have respiratory mechanics that match those of patients with classic ARDS, with a large unimodal distribution of respiratory system compliance (C_RS_).^(12-14)^ These findings suggest that protective ventilatory strategies are recommended for patients with COVID-19-related ARDS and that, at the bedside, C_RS_ might be considered to personalize ventilatory support.

Traditionally, protective MV is based on limiting V_T_ (4 - 8mL/kg predicted body weight [PBW]) and P_plat_ (30cmH_2_O).^(9)^ However, adjusting the V_T_ based on the PBW does not consider lung heterogenicity in ARDS patients. In severe forms of ARDS, only a small proportion of the lung is available for ventilation (the "baby lung" concept).^(15)^ Therefore, for a "baby lung", even a low V_T_ calculated for the PBW might be injurious.^(16)^

Alternatively, the V_T_ can be set considering the extent of lung involvement, which can be inferred at the bedside by the C_RS_. Indeed, Amato et al. demonstrated that the DP, which represents the V_T_ normalized to the C_RS_, was the ventilatory parameter most strongly associated with survival. In addition, they showed that V_T_ normalized to the PBW was not a strong predictor of survival.^(6)^ These findings suggest that it might be better to scale V_T_ to the aerated lung available for ventilation than to the lung size.

Therefore, we hypothesize that among COVID-19 ARDS patients, DP, as opposed to V_T_ based on the PBW, is associated with survival. The aim of our study was to evaluate the association between DP or V_T_ and mortality in a cohort of COVID-19 ARDS patients.

## METHODS

### Study population

This was a prospective cohort study conducted between April 2020 and June 2021 in two COVID-19 dedicated intensive care units (ICUs) in Juiz de Fora (Minas Gerais, Brazil): *Hospital Universitário* of the *Universidade Federal de Juiz de Fora* (13 beds) and *Hospital Regional Dr. João Penido* (20 beds). The study followed the principles of the Declaration of Helsinki and was approved by the *Hospital Universitário* of the *Universidade Federal de Juiz de Fora* Ethics Committee and National Research Ethics Committee (CAAE: 30282920.5.1001.5133). Close relatives of the patients provided written informed consent.

All consecutive patients aged 18 years or older who were admitted to one of the participating ICUs with COVID-19 confirmed by reverse transcription polymerase chain reaction (RT-PCR) were eligible for participation if they had ARDS according to the Berlin criteria and were invasively ventilated.^(1)^ Patients who were ventilated for more than 24 hours before admission to the participant ICU and those for whom life-sustaining treatment was withheld were excluded.

### Data collection

At admission to the ICU, we collected demographic and clinical data and calculated the following scores: Simplified Acute Physiology Score 3 (SAPS 3), Sequential Organ Failure Assessment (SOFA), and Charlson comorbidity index. The SOFA score was also calculated for the first three days of MV.

We defined Day 0 (D0) as the calendar day when a patient was intubated. The following ventilatory parameters were collected at D0 after stabilization of the patient: ventilatory mode, V_T_, respiratory rate (RR), positive end-expiratory pressure (PEEP), P_plat_, DP (P_plat_ minus total PEEP), and C_RS_ (V_T_ divided by DP). The V_T_ was also expressed as V_T_ normalized to the PBW (mL/kg PBW). The PBW was calculated by the following equation: PBW = 50 + 0.91 × (height expressed in cm – 152.4), for males; PBW = 45.5 + 0.91 × (height expressed in cm – 152.4), for females.^(17)^ Arterial blood gas analysis was performed simultaneously with the ventilatory parameters. The same ventilatory parameters were collected on Days 1 and 2, as close to 8 a.m. as possible.

Both ICUs adopted a protocol of protective MV. The initial ventilatory mode was either volume-controlled ventilation or pressure-controlled ventilation, adjusted to offer a V_T_ of 6mL/kg PBW. The V_T_ was reduced to 4 - 5mL/kg PBW to ensure that the DP was ≤ 15cmH_2_O. In these patients, to maintain the same minute of ventilation recorded before the reduction in V_T_, the RR was increased to 35 breaths/min. Conversely, a V_T_ of up to 8mL/kg PBW was allowed to improve patient-ventilator synchrony or to correct acidosis, provided that the DP was ≤ 15cmH_2_O.

Positive end-expiratory pressure and fraction of inspired oxygen (FiO_2_) were adjusted according to the ARDSnet table (low PEEP table) to maintain oxygen saturation (SpO_2_) between 93% and 96%.^(17)^ Patients with a ratio of arterial oxygen partial pressure to fraction of inspired oxygen (PaO_2_/FiO_2_) < 150mmHg with a PEEP ≥ 10cmH_2_O were switched to the prone position and ventilated in this position for 16-18 hours. The criteria for stopping prone treatment were as follows: (1) improvement in oxygenation (PaO_2_/FiO_2_ ≥ 150mmHg with PEEP ≤ 10cm H_2_O, four hours after the end of the prone session); (2) decrease in the PaO_2_/FiO_2_ >20% in the prone position relative to the ratio in the supine position; and (3) complications occurring during the prone session. If prone positioning was contraindicated or if it was ineffective at increasing PaO_2_/FiO_2_, a recruitment maneuver with incremental PEEP levels (up to 25cm H_2_O), followed by a decremental PEEP titration according to the best C_RS_ was performed. Extracorporeal membrane oxygenation and inhaled nitric oxide were not available in the participating ICUs.

The ventilator mode was switched to pressure support in alert patients when the PEEP was ≤ 10cmH_2_O and the patients were awake enough to breathe in this mode. The pressure support level was started with the level necessary to achieve a V_T_ of 6 to 8mL/kg PBW and an RR of 12 to 30 respirations per minute (rpm). Pressure support was reduced in 2cmH_2_O every 2 – 4 hours provided that the patient remained comfortable, with a V_T_ of 6 to 8mL/kg PBW and an RR of 12 - 30rpm. A patient was assumed to be ready for extubation if they presented PaO_2_/FiO_2_ > 200mmHg and an RR between 8 and 30rpm with no signs of respiratory distress for at least 30 minutes while ventilated with a pressure support of 7cmH_2_O, PEEP ≤ 8cm H_2_O, and FiO_2_ ≤0.4, providing that he or she was clinically stable. Patients ventilated for more than 14 days were considered for tracheostomy if they presented one of the following indications: MV expected to last at least seven days more, Glasgow coma score lower than 8, inadequate swallowing or cough reflex with retention of sputum. Weaning with a tracheostomy followed the same protocol described above.

### Outcomes

The primary outcome was hospital mortality. Secondary outcomes included 28-day mortality, ICU mortality, duration of MV and length of stay in the ICU and hospital.

### Statistical analysis

The sample size was based on the number of patients admitted to the participating ICUs who met the inclusion criteria and constituted a convenience sample. Therefore, these findings should be interpreted as exploratory.

Continuous variables are reported as medians (quartile 25% - quartile 75%) and were compared with the Wilcoxon rank-sum test, and categorical variables are reported as numbers and relative proportions and were compared with the chi-square test. The V_T_/kg PBW, P_plat_ and DP on Days 1 and 2 are presented in cumulative distribution plots.

**Figure 1:**
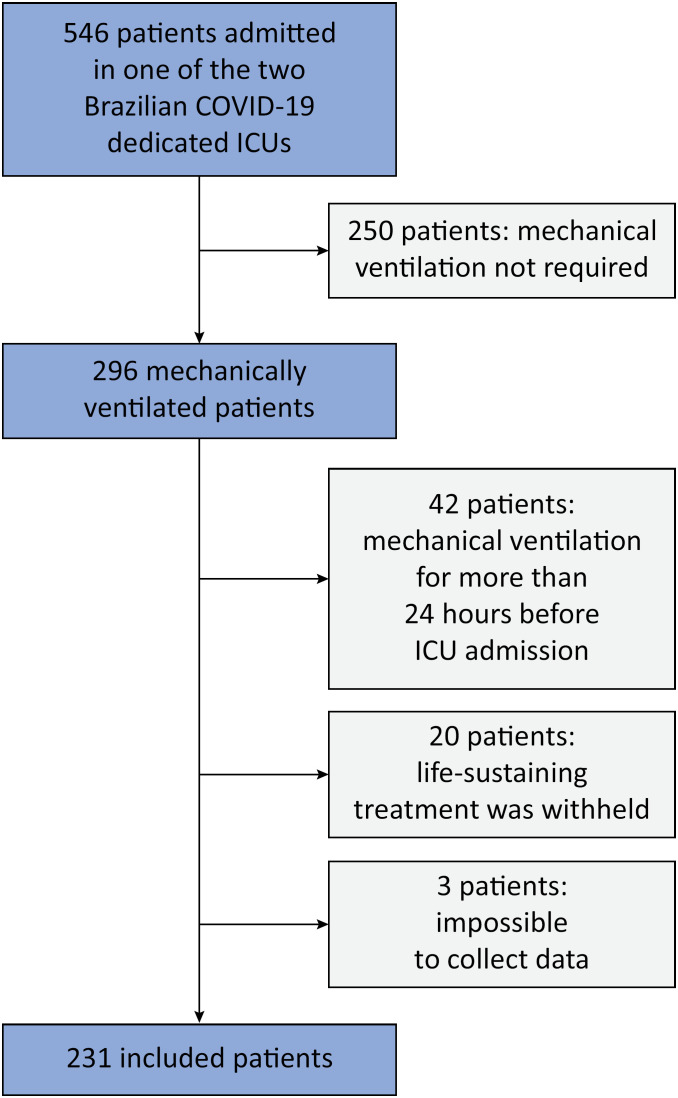
Study participant flow chart.

**Figure 2:**
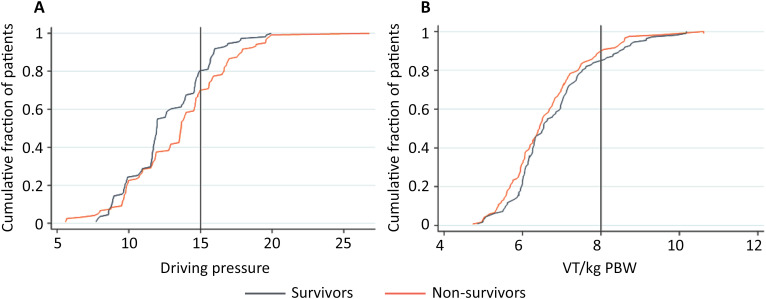
Ventilatory parameters on the first day of mechanical ventilation.

Multivariable logistic models were used to evaluate whether V_T_/kg PBW and DP were independently associated with hospital mortality. In both models, confounders were selected using a directed acyclic graph (DAG) (Figures 1S and 2S - Supplementary Material). In the model with V_T_/kg PBW as the independent variable of interest, the selected confounders were sex, C_RS_, DP, and RR. In the model with DP as the independent variable of interest, the selected confounders were C_RS_ and V_T_/kg PBW.

In addition, we performed a double stratification analysis to evaluate the association between DP and in-hospital mortality when V_T_/kg PBW was kept constant. First, we created six quantiles of V_T_/kg PBW. Second, each quantile was stratified into three quantiles of increasing DP. Third, for each stratum of V_T_/kg PBW, we merged the three clusters of DP, forming three subsamples with matched average V_T_/kg PBW and increasing average DP. We repeated the double stratification analysis to obtain three subsamples with matched average DP and increasing average V_T_/kg PBW.

All analyses were conducted with Stata 15.1 (Stata CorpLP, College Statio, TX, USA), and the significance level was set at 0.05.

## RESULTS

During the study period, 546 patients were admitted to the participating ICUs, and 296 met the inclusion criteria. Among those patients, 65 were excluded, and 231 constituted the final cohort. The main reasons for exclusion were that they were invasively ventilated for more than 24 hours before admission to the ICU and that they had been started on palliative care by the treatment team (Figure 1).

ICU - intensive care unit.

The patients had a median age of 64 years (IQR 53 - 74), 52.8% (122 patients) were male, the median SAPS 3 score was 45 (IQR 39 - 54), 56.2% (121 patients) had moderate ARDS, and 19.5% (42 patients) had severe ARDS. The most prevalent comorbidities were hypertension and diabetes (Table 1). The hospital mortality rate was 51.9% (120 patients). Patients who survived were younger and had lower SAPS-3, SOFA, and Charlson comorbidity index scores. Diabetes, cardiovascular disease, and chronic kidney disease were more prevalent among nonsurvivors (Table 1). Patients who survived had a lower DP and greater C_RS_ but similar V_T_/kg PBW, P_plat_, P_peak_, and PEEP (Table 2, Figure 2). The PaO_2_/FiO_2_ ratio and pH were greater in patients who survived (Table 2).

**Table 1:** Baseline demographic and clinical characteristics of patients

		All (n = 231)	Survivors (n = 111)	Nonsurvivors (n = 120)	p value
Age (years)		64 (53 -74)	57 (46 - 65)	70 (60 - 78)	< 0.0001
Male		122 (52.8)	54 (48.6)	68 (56.6)	0.22
PBW (kg)		61.4 (52.4 - 67.8)	59.7 (52.4 - 66.0)	61.4 (52.4 - 70.5)	0.47
SAPS 3		45 (39 - 54)	41 (38 - 47)	51 (43 - 60)	< 0.0001
SOFA		3 (2 - 6)	3 (2 - 4)	4 (2 - 8)	0.0001
Charlson comorbidity index		3 (1 - 5)	2 (1 - 3)	4 (2 - 5)	< 0.0001
Comorbidities					
	Cardiovascular disease	36 (15.8)	11 (9.9)	25 (20.8)	0.02
	Chronic kidney disease	17 (7.3)	4 (3.6)	13 (10.8)	0.03
	Chronic pulmonary disease	26 (11.2)	10 (9.0)	16 (3.3)	0.29
	Diabetes	91 (39.3)	33 (29.7)	58 (48.3)	0.004
	Hypertension	152 (65.8)	66 (59.4)	86 (71.6)	0.051
	Obesity	48 (20.7)	27 (24.3)	21 (17.5)	0.20
ARDS severity					0.10
	Mild	52 (24.1)	30 (28.5)	22 (20.0)	
	Moderate	121 (56.2)	60 (57.1)	61 (55.4)	
	Severe	42 (19.5)	15 (14.2)	27 (24.5)	

PBW - predicted body weight; SAPS - Simplified Acute Physiology Score; SOFA - Sequential Organ Failure Assessment; ARDS - acute respiratory distress syndrome. Categorical variables are expressed as n (%), and continuous variables are expressed as medians (interquartile ranges).

Cumulative frequency distribution of driving pressure, cmH_2_O (A), and tidal volume, mL/kg predicted body weight (B). Vertical lines represent the respective cutoffs of driving pressure and tidal volume for protective mechanical ventilation. V_T_ - tidal volume; PBW - predicted body weight.

**Table 2:** Respiratory parameters on Day 1 of mechanical ventilation and clinical outcomes

		All (n = 231)	Survivors (n = 111)	Nonsurvivors (n = 120)	p value
Mode of ventilation					0.15
	Pressure-controlled ventilation	134 (58.0)	58 (52.2)	76 (63.3)	
	Volume-controlled ventilation	96 (41.5)	52 (46.8)	44 (36.6)	
	Pressure-support ventilation	1 (0.4)	1 (0.90)	0	
Tidal volume (mL/kg PBW)		6.48 (5.96 - 7.21)	6.54 (6.01 - 7.38)	6.43 (5.90 - 7.13)	0.17
Peak pressure (cmH_2_O)		26 (24 - 29)	26 (24 - 29)	26 (24 - 30)	0.68
Plateau pressure (cmH_2_O)		24 (21 - 27)	24 (21 - 26)	24 (22 - 28)	0.39
Driving pressure (cmH_2_O)		13 (11 - 16)	12 (11 - 15)	14 (11 - 16)	0.03
PEEP (cmH_2_O)		10 (10 - 12)	10 (10 - 12)	10 (10 - 12)	0.63
C_RS_ (mL/cmH_2_O)		30.2 (24.6 - 36.6)	31.7 (26.2 - 36.6)	28.6 (23.3 - 35.0)	0.02
Prone ventilation*		134 (58.0)	60 (54.0)	74 (61.6)	0.24
FiO_2_		0.60 (0.50 - 0.75)	0.60 (0.45 - 0.70)	0.60 (0.50 - 0.80)	0.04
PaO_2_/FiO_2_ (mmHg)		195 (147.1 - 253.3)	210 (157.7 - 260)	187 (137.8 - 241.6)	0.06
PaCO_2_ (mmHg)		45 (40 - 53)	45 (40 - 53)	45 (39.4 - 52.9)	0.84
pH		7.34 (7.28 - 7.40)	7.35 (7.28 - 7.41)	7.33 (7.27 - 7.37)	0.01
Hemodialysis		63 (27.7)	17 (15.3)	46 (38.3)	< 0.0001
Duration of MV (days)		13 (7 - 24)	10 (6 - 21)	15 (7 - 26)	0.07
Length of ICU stay (days)		16 (9 - 28)	16 (10 - 28)	16 (7 - 29)	0.39
Length of hospital stay (days)		22 (12 - 38)	27 (18 - 50)	16 (7 - 31.5)	< 0.0001

PBW - predicted body weight; PEEP - positive end expiratory pressure; C_RS_ - compliance of the respiratory system; FiO_2_ - fraction of inspired oxygen; PaO_2_/FiO_-_ ratio of arterial oxygen partial pressure to fraction of inspired oxygen; PaCO_2_ - arterial carbon dioxide partial pressure; MV - mechanical ventilation; ICU - intensive care unit.

* Patients under prone ventilation at any time during mechanical ventilation. Categorical variables are expressed as n (%), and continuous variables are expressed as medians (interquartile ranges).

According to the multivariate analysis, DP was independently associated with in-hospital mortality. Nevertheless, no statistically significant association was found between V_T_/kg PBW and in-hospital mortality (Table 3). Based on the double stratification analysis, we found that for the same level of V_T_/kg PBW, the risk of hospital death increased with increasing DP (Table 4). However, for the same DP, the risk of hospital death did not increase with increasing V_T_/kg PBW (Table 4).

**Table 3:** Multivariable logistic regression assessing the association between tidal volume or driving pressure and hospital mortality

		Odds ratio (95%CI)	p value
V_T_/kg PBW		0.75 (0.55-1.04)	0.09
	Female	0.91 (0.45-1.85)	0.80
	C_RS_	1.03 (0.97-1.10)	0.30
	DP	1.20 (1.01-1.43)	0.03
	RR	1.01 (0.95-1.08)	0.62
DP		1.21 (1.04-1.41)	0.01
	C_RS_	1.03 (0.98-1.08)	0.15
	V_T_/kg PBW	0.74 (0.56-0.96)	0.02

95%CI - 95% confidence interval; V_T_ - tidal volume; PBW - predicted body weight; C_RS_ - compliance of the respiratory system; DP - driving pressure; RR - respiratory rate. The associations between tidal volume/kg predicted body weight and hospital mortality were adjusted for sex, compliance of the respiratory system, driving pressure and respiratory rate. The association between driving pressure and hospital mortality was adjusted for compliance of the respiratory system and tidal volume/kg predicted body weight.

**Table 4:** Double stratification analysis: impact of tidal volume/kg predicted body weight when driving pressure is kept constant and of driving pressure when tidal volume/kg predicted body weight is kept constant

	Multivariable analysis considering quantile 1 as reference (Three quantiles of increasing V_T_/kg PBW, with constant DP)		
	Quantile 1 (n = 76)	Quantile 2 (n = 77)	Quantile 3 (n = 78)
DP	13 (11 - 15)	14 (11 - 15)	13 (11 - 16)
V_T_/kg PBW	5.7 (5.4 - 6.0)	6.5 (6.3 - 6.8)	7.7 (7.2 - 8.5)
Hospital mortality %	57	58	41
Odds ratio (95%CI)		1.13 (0.59 - 2.20)	0.58 (0.30 - 1.12)
p value		0.71	0.11
	Multivariable analysis considering quantile 1 as reference (Three quantiles of increasing DP, with constant V_T_/kg PBW)		
	Quantile 1 (n = 94)	Quantile 2 (n = 71)	Quantile 3 (n = 66)
V_T_/kg PBW	6.5 (5.9 - 7.1)	6.6 (6.0 - 7.4)	6.4 (6.0 - 7.4)
DP	10 (9 - 12)	14 (13 - 15)	17 (16 - 18)
Hospital mortality %	44	56	59
Odds ratio (95%CI)		1.72 (0.92 - 3.25)	1.93 (1.02 - 3.71)
p value		0.09	0.046

V_T_ - tidal volume; PBW - predicted body weight; DP - driving pressure; 95%CI - 95% confidence interval; DP - driving pressure. Multivariate analyses adjusted for sex and respiratory rate.

Regarding the secondary outcomes, at the end of the 28-day follow-up, 86 (37.2%) patients had died. The ICU mortality rate was 47.6% (110 patients). The median ICU stay was 16 (IQR 9 - 28) days, and the median hospital stay was 22 (12 - 38) days.

## DISCUSSION

This observational study evaluated a cohort of patients with COVID-19 who met the ARDS criteria and underwent invasive MV. DP, which represents V_T_ normalized for C_RS_, was independently associated with in-hospital mortality. However, when the V_T_ was adjusted for PBW, no statistically significant association with hospital mortality was identified.

Since 2000, when the ARMA Study showed that ventilating ARDS patients with lower V_T_ and lower P_plat_ decreases mortality,^(17)^ guidelines on the management of ARDS have recommended that these patients receive MV with limited V_T_ (4 - 8mL/kg PBW) and P_plat_ (lower than 30cmH_2_O).^(9)^ Despite the benefits of ventilating ARDS patients with low V_T_, especially when compared with V_T_ of 12mL/kg PBW, adjusting the V_T_ only by the PBW has been criticized by some authors because this strategy does not consider the severity of ARDS or the amount of lung tissue that is ventilated.^(16,18)^ For example, Terragni et al., analyzing ARDS patients with computed tomography, demonstrated that, in patients with large nonaerated areas (characterizing a "small baby lung"), lowering V_T_ to 6mL/kg PBW and limiting P_plat_ to 30cmH_2_O may not be sufficient to avoid hyperinflation and to minimize VILI.^(16)^

An alternative strategy to minimize VILI in ARDS patients is to normalize V_T_ to C_RS_ by targeting DP (DP = V_T_/C_RS_) as a protective parameter. Since the stress and strain that lead to VILI are determined both by V_T_ and end-expiratory lung volume, the latter determined by the amount of aerated tissue and represented by the C_RS_, DP might be a better predictor of mortality than V_T_ or P_plat_.^(19)^ The DP can be easily calculated at the bedside, and several observational studies have shown its association with mortality.^(20,21)^ Moreover, Amato et al., analyzing data from ARDS patients enrolled in a previous randomized trial that compared different ventilatory strategies, showed that higher DP predicted lower survival, whereas higher P_plat_ and V_T_ were associated with lower survival only in patients with elevated DP.^(6)^

Our results, obtained among COVID-19 patients who developed ARDS, are consistent with those showing that DP is a better predictor of mortality than V_T_/kg PBW. In our cohort, a higher DP, as opposed to a higher V_T_ based on the PBW, was associated with greater hospital mortality. In addition, at constant levels of V_T_/kg PBW, we observed that DP was associated with mortality, while at constant levels of DP, an increase in V_T_/kg PBW was not associated with a greater risk of death. In fact, in our cohort, we showed a lower risk of death with increasing V_T_/kg PBW, although the difference was not statistically significant. This finding might reflect the strategy of setting the ventilatory parameters. Since we used our protective MV to maintain the DP lower than 15cmH_2_O, patients with lower C_RS_ may have been ventilated with lower V_T_/kg PBW, resulting in an association between lower V_T_/kg PBW and higher mortality. However, we cannot exclude the possibility that V_T_/kg PBW reduction in patients with higher C_RS_ is not harmful by itself, as it increases the risk of asynchrony and the necessity of deep sedation and neuromuscular blockade.^(22,23)^ This impact of respiratory mechanics on the effect of lowering V_T_ in ARDS patients was recently demonstrated. The authors showed that the effect of decreasing V_T_ on mortality varied according to C_RS_ and that the difference in the risk of death between patients with higher and lower V_T_ was statistically significant only in patients with low C_RS_.^(24)^

Our study has several relevant limitations. The cohort was formed in only two dedicated COVID-19 ICUs that followed the same protocol for ventilatory management. Therefore, the results may not be generalizable to other ICUs. Ventilatory parameters were collected only on the first three days of MV and may not represent those applied during the following days. We cannot exclude the possibility that ventilatory parameters and adjuvant therapies applied beyond Day 3 influenced mortality. As is common in observational studies, the knowledge of ICU teams about the study being conducted could have interfered with the way patients were ventilated, increasing adherence to protective MV. Over the 15 months of the study, changes in the management, not related to MV, of patients with COVID-19, as well as the experience acquired by the ICU teams, may have influenced the survival of these patients, and the causal model might not have been able to identify them. We do not have data regarding ventilatory support before intubation (high-flow nasal cannula or noninvasive ventilation). Therefore, the possible effects of these treatments could not be assessed in this study. This was an observational study, and the analyzed relationships (C-ARDS cohort with protective MV and protective MV with hospital mortality) might have been influenced by residual confounding factors not included in the DAGs. Therefore, causality cannot be assured.

## CONCLUSION

In our cohort of acute respiratory distress syndrome patients with COVID-19, exposure to greater driving pressure was associated with greater hospital mortality. However, there was no significant association between tidal volume adjusted for predicted body weight and hospital mortality. These results suggest that lung-protective mechanical ventilation might primarily target driving pressure rather than tidal volume adjusted by predicted body weight.

## Supplementary Material


