# Supplementary material for: Driving pressure, as opposed to tidal volume based on predicted body weight, is associated with mortality: results from a prospective cohort of COVID-19 acute respiratory distress syndrome patients
Source: Crit Care Sci. 2024 Apr 22;36:e20240208en. doi: 10.62675/2965-2774.20240208-en (PMC11098065; doi:10.62675/2965-2774.20240208-en)
Supplement: Supplementary file 1 [file 2965-2774-ccsci-36-e20240208en-Suppl01.pdf]

Critical Care Science
https://doi.org/10.62675/2965-2774.20240208-en

SUPPLEMENTARY MATERIAL

Driving pressure, as opposed to tidal volume based on predicted body weight, is associated with mortality: results from a prospective cohort of COVID-19 acute respiratory distress syndrome patients

Erich Vidal Carvalho[1], Maycon Moura Reboredo[1], Edimar Pedrosa Gomes[1], Pedro Nascimento Martins[1], Gabriel Paz Souza Mota[1], Giovani Bernardo Costa[1], Fernando Antonio Basile Colugnati[1], Bruno Valle Pinheiro[1]

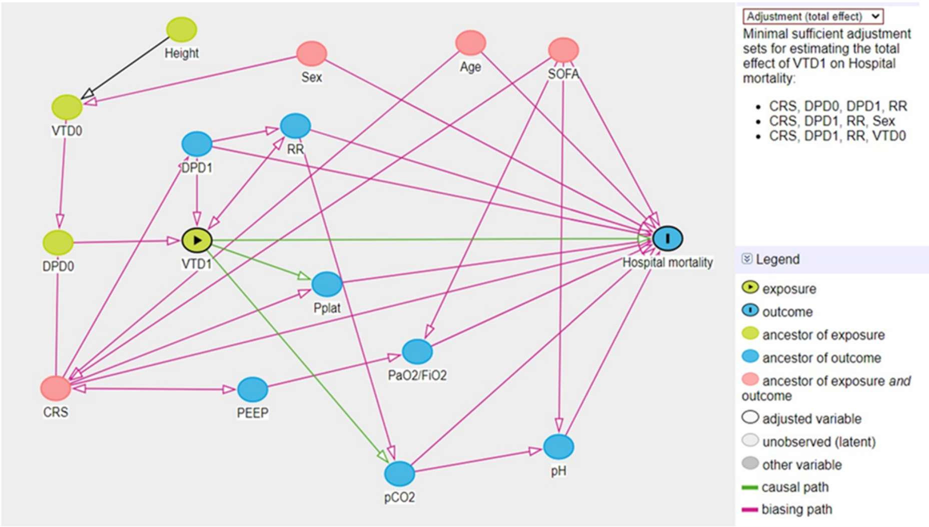

Figure 1S - Directed acyclic graph built with variables that are biologically associated with exposure (tidal volume/kg predicted body weight on day 1 of mechanical ventilation) and/or outcome (hospital mortality), according to previous studies.

The assumptions are based on previous knowledge (for example, values of compliance of the respiratory system can be associated with both tidal volume/kg predicted body weight and hospital mortality) or in the absence of data supporting the exclusion of any possible association. Based on these assumptions, the adjustment necessary for estimating the effect of tidal volume/kg predicted body weight on hospital mortality, according to the dagitty tool, includes the following variables: sex, driving pressure, compliance of the respiratory system and respiratory rate.

Green circle with black line: exposure; blue circle with black line: outcome; pink circle: ancestor of exposure and outcome; blue circle: ancestor of outcome; green circle: ancestor of outcome; gray circle: unobserved (latent).

C_{RS} - compliance of the respiratory system; DP0 - driving pressure on day 0 of mechanical ventilation; DP1 - driving pressure on day 1 of mechanical ventilation; $PaCO_2$ - arterial carbon dioxide partial pressure; PaO_2/FiO_2 - ratio of arterial oxygen partial pressure to fraction of inspired oxygen; PEEP - positive end expiratory pressure; P_{plat} - plateau pressure; RR - respiratory rate; V_TD0 - tidal volume on day 0 of mechanical ventilation; V_TD1 - tidal volume on day 1 of mechanical ventilation; SOFA - Sequential Organ Failure Assessment.

This is an open access article under the CC BY license (https://creativecommons.org/licenses/by/4.0/).

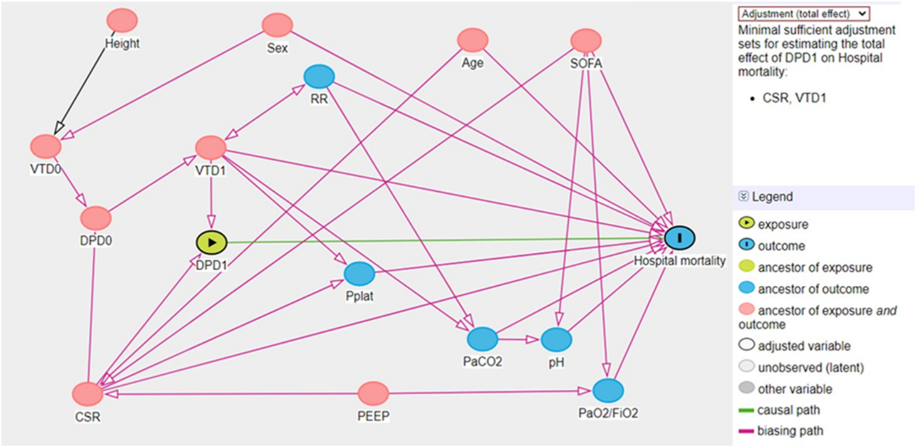

Figure 2S - Directed acyclic graph built with variables that are biologically associated with exposure (driving pressure on day 1 of mechanical ventilation) and/or outcome (hospital mortality), according to previous studies.

The assumptions are based on previous knowledge (for example, values of compliance of the respiratory system can be associated with both driving pressure and hospital mortality) or in the absence of data supporting the exclusion of any possible association. Based on these assumptions, the adjustment necessary for estimating the effect of driving pressure on hospital mortality, according to the dagitty tool, includes the following variables: compliance of the respiratory system and tidal volume on day 1 of mechanical ventilation.

Green circle with black line: exposure; blue circle with black line: outcome; pink circle: ancestor of exposure and outcome; blue circle: ancestor of outcome; green circle: ancestor of outcome; gray circle: unobserved (latent).

CRS - compliance of the respiratory system; DP0 - driving pressure on day 0 of mechanical ventilation; DP1 - driving pressure on day 1 of mechanical ventilation; $PaCO_2$ - arterial carbon dioxide partial pressure; PaO_2/FiO_2 - ratio of arterial oxygen partial pressure to fraction of inspired oxygen; PEEP - positive end expiratory pressure; P_{plat} - plateau pressure; RR - respiratory rate; V_TD0 - tidal volume on day 0 of mechanical ventilation; V_TD1 - tidal volume on day 1 of mechanical ventilation; SOFA - Sequential Organ Failure Assessment.
