# Supplementary material for: Driving pressure, as opposed to tidal volume based on predicted body weight, is associated with mortality: results from a prospective cohort of COVID-19 acute respiratory distress syndrome patients
Source: Crit Care Sci. 2024 Apr 22;36:e20240208en. doi: 10.62675/2965-2774.20240208-en (PMC11098065; doi:10.62675/2965-2774.20240208-en)
Supplement: Translation

# Translation: *Driving pressure*, em oposição ao volume corrente ajustado para o peso corporal predito, associa-se com a mortalidade: resultados de uma coorte prospectiva de pacientes com síndrome do desconforto respiratório agudo por COVID-19

**DOI:** 10.62675/2965-2774.20240208-pt

**Authors:** Erich Vidal Carvalho, Maycon Moura Reboredo, Edimar Pedrosa Gomes, Pedro Nascimento Martins, Gabriel Paz Souza Mota, Giovani Bernardo Costa, Fernando Antonio Basile Colugnati, Bruno Valle Pinheiro

**Affiliations:** 1 Juiz de Fora MG Brasil Serviço de Terapia Intensiva e Pneumologia, Hospital Universitário, Universidade Federal de Juiz de Fora - Juiz de Fora (MG), Brasil.

**Keywords:** Síndrome do desconforto respiratório agudo, COVID-19, Infecções por coronavírus, Volume corrente, Respiração artificial, Mortalidade, Unidades de terapia intensiva

## Abstract

**Objetivo:**

Avaliar a associação entre *driving pressure* e volume corrente ajustado pelo peso predito com a mortalidade em uma coorte de pacientes com síndrome do desconforto respiratório agudo por COVID-19.

**Métodos:**

Estudo prospectivo e observacional que incluiu pacientes com síndrome do desconforto respiratório agudo por COVID-19 admitidos em duas unidades de terapia intensiva. Foi realizada análise multivariada para determinar se a *driving pressure* e o volume corrente/kg de peso predito, aferidos no primeiro dia de ventilação mecânica, associavam-se de forma independente com a mortalidade hospitalar.

**Resultados:**

Foram incluídos 231 pacientes. A mediana de idade foi de 64 (53 - 74) anos, e a mediana do *Simplified Acute and Physiology Score 3* foi de 45 (39 - 54). A mortalidade hospitalar foi de 51,9%. A *driving pressure* se associou de forma independente com a mortalidade hospitalar (razão de chance de 1,21; IC95% de 1,04 - 1,41 para cada cm H_2_O de aumento da *driving pressure*, p = 0,01). Com base na análise de dupla estratificação, encontrou-se que, para o mesmo nível de volume corrente/kg de peso predito, o risco de mortalidade hospitalar aumentava com o incremento da *driving pressure*. No entanto, mudanças no volume corrente/kg de peso predito não se associaram com a mortalidade quando não resultavam em aumento da *driving pressure*.

**Conclusão:**

Em pacientes com síndrome do desconforto respiratório agudo por COVID-19, exposição a maior *driving pressure*, ao contrário da exposição a maior volume corrente/kg de peso predito, associou-se com maior mortalidade hospitalar. Os resultados sugerem que a *driving pressure* poderia ser o alvo primário para a condução da ventilação mecânica protetora nesses pacientes.

## INTRODUÇÃO

Pacientes com insuficiência respiratória aguda hipoxêmica por COVID-19 frequentemente preenchem os critérios diagnósticos da síndrome do desconforto respiratório agudo (SDRA), conforme a definição de Berlim, e necessitam de ventilação mecânica.^(1-3)^ Apesar de essencial para o tratamento de pacientes com SDRA de diferentes etiologias, inclusive a COVID-19, a ventilação mecânica (VM) pode agravar a lesão inflamatória pulmonar já instalada, um fenômeno conhecido como lesão pulmonar induzida pelo ventilador mecânico (LPIV).^(4)^ Diversos estudos conduzidos em pacientes com SDRA mostraram associação entre diferentes variáveis ventilatórias e a mortalidade, como volume corrente (V_T_), pressão de platô (P_plat_), *driving pressure* (DP) e *mechanical power* (MP).^(5-8)^ Com base nesses estudos, os ajustes ventilatórios devem ter como objetivo não apenas a correção da hipoxemia, mas também proteger os pulmões da LPIV.^(9)^

No início da pandemia, dois diferentes fenótipos foram descritos entre os pacientes com COVID-19 que desenvolveram insuficiência respiratória aguda hipoxêmica. Enquanto alguns pacientes tiveram uma apresentação típica da SDRA, com alta elastância, alto peso pulmonar e maior potencial para a realização de recrutamento alveolar, outros indivíduos, para o mesmo nível de hipoxemia, apresentavam baixa elastância e baixo peso pulmonar, com menor potencial para o recrutamento alveolar.^(10,11)^ Naquele momento, não estava claro se os pacientes com SDRA por COVID-19 deveriam ser ventilados seguindo os mesmos princípios recomendados para SDRA de outras etiologias. No entanto, posteriormente, vários estudos mostraram que pacientes com SDRA por COVID-19 apresentavam mecânica respiratória semelhante com aqueles com SDRA clássica, com ampla distribuição unimodal da complacência do sistema respiratório (C_SR_).^(12-14)^ Esses achados sugerem que a estratégia ventilatória protetora deva ser empregada na SDRA por COVID-19 e que, a beira do leito, a C_SR_ poderia ser considerada na individualização dos ajustes ventilatórios.

Tradicionalmente, a VM protetora se baseia na limitação do V_T_ (4 - 8mL/kg de peso corporal predito [PCP]) e na P_plat_ (30cmH_2_O).^(9)^ Entretanto, ajustar o V_T_ baseando no PCP não considera a heterogeneidade do pulmão de pacientes com SDRA. Nas formas graves da SDRA, apenas uma pequena proporção do pulmão está disponível para a ventilação (o conceito de *baby lung*).^(15)^ Assim, para um *baby lung*, mesmo um baixo V_T_, calculado pelo PCP, pode ser prejudicial.^(16)^

Alternativamente, o V_T_ pode ser ajustado considerando a extensão do acometimento pulmonar, que pode ser inferido a beira do leito pela C_SR_. De fato, Amato et al. demonstraram que a DP, que representa o V_T_ normalizado pela C_SR_, foi a variável ventilatória mais fortemente associada com a sobrevida de pacientes com SDRA. Além disso, eles mostraram que o V_T_ ajustado pelo PCP não foi um forte preditor de sobrevida nessa população.^(6)^ Esses achados sugerem que poderia ser melhor ajustar o V_T_ de acordo com a área de pulmão aerado disponível do que se basear no tamanho do pulmão.

Nossa hipótese foi a de que, em pacientes com SDRA por COVID-19, a DP, ao contrário do V_T_ ajustado pelo PCP, estaria associada à sobrevida. O objetivo do estudo foi avaliar a associação entre DP e V_T_ com mortalidade em uma coorte de pacientes com SDRA por COVID-19.

## MÉTODOS

### População do estudo

Este é um estudo de coorte prospectiva conduzido no período de abril de 2020 a junho de 2021, em duas unidades de terapia intensiva (UTIs) dedicadas ao atendimento de pacientes com COVID-19 em Juiz de Fora (MG): o Hospital Universitário da Universidade Federal de Juiz de Fora (13 leitos) e o Hospital Regional Dr. João Penido (20 leitos). O estudo seguiu os princípios da Declaração de Helsinki e foi aprovado pelo Comitê de Ética da Universidade Federal de Juiz de Fora e pela Comissão Nacional de Ética em Pesquisa (CAAE: 30282920.5.1001.5133). Parentes próximos dos pacientes assinaram o Termo de Consentimento Livre e Esclarecido.

Foram elegíveis para o estudo todos os pacientes maiores de 18 anos admitidos de forma consecutiva nas UTIs participantes e que tivessem diagnóstico confirmado de COVID-19 pelo exame de reação em cadeia da polimerase via transcriptase reversa (RT-PCR), necessidade de VM invasiva e preenchessem os critérios diagnósticos de Berlim para a SDRA.^(1)^ Foram excluídos os indivíduos ventilados por mais de 24 horas antes da admissão na UTI e aqueles com indicação de cuidados paliativos pela equipe assistente.

### Coleta de dados

Na admissão na UTI, foram coletados os dados demográficos e clínicos e calculados os seguintes escores: *Simplified Acute and Physiology Score 3* (SAPS 3), *Sequential Organ Failure Assessment* (SOFA) e o índice de comorbidades de Charlson. O SOFA também foi calculado nos três primeiros dias de VM.

Foi definido como Dia 0 (D0) de VM o dia do calendário no qual o paciente foi intubado. Os seguintes parâmetros foram coletados no D0, após a estabilização do paciente: modo ventilatório, frequência respiratória (FR), pressão expiratória positiva final (PEEP), P_plat_, DP (P_plat_ menos PEEP total) e C_SR_ (V_T_ dividido pela DP). O V_T_ foi expresso conforme o PCP (mL/kg PCP), calculado pelas seguintes equações: PCP = 50 + 0,91 × (altura em centímetros – 152,4), para os homens e PCP = 45,5 + 0,91 × (altura em centímetros – 152,4), para as mulheres.^(17)^ A gasometria arterial foi analisada simultaneamente com os parâmetros ventilatórios. Os mesmos parâmetros ventilatórios foram coletados nos Dias 1 e 2 de VM, próximo às 8h.

As UTIs participantes adotavam o protocolo de VM protetora. O modo ventilatório inicial, seja volume-controlado ou pressão-controlada, era ajustado para oferecer um V_T_ de 6mL/kg PCP. O V_T_ poderia ser posteriormente reduzido para 4 - 5mL/kg PCP para garantir uma DP ≤ 15cmH_2_O. Nessa situação, para manter o mesmo volume-minuto antes do ajuste do V_T_, a FR poderia ser aumentada até 35 incursões respiratórias por minuto (irpm). Por outro lado, o V_T_ poderia ser aumentado até 8mL/kg PCP para permitir melhora da sincronia paciente-ventilador ou para correção de acidose, desde que a DP permanecesse < 15cmH_2_O.

Pressão expiratória positiva final e fração inspirada de oxigênio (FiO_2_) eram ajustadas de acordo com a tabela PEEP/FiO_2_ do ARDSnet (*low* PEEP *table*) para manter a saturação de oxigênio (SpO_2_) entre 93% e 96%.^(17)^ Pacientes com relação entre a pressão parcial de oxigênio e a fração inspirada de oxigênio (PaO_2_/FiO_2_) < 150mmHg e PEEP ≥ 10cmH_2_O eram colocados em posição prona e ventilados por 16 a 18 horas nessa posição. Os critérios para interromper a ventilação em posição prona eram: melhora da oxigenação (PaO_2_/FiO_2_ ≥ 150mmHg com PEEP < 10cmH_2_O, por 4 horas após o fim da sessão de prona); redução da PaO_2_/FiO_2_ > 20% em posição prona quando comparada com a posição supina; ocorrência de complicações durante a sessão de prona. Caso houvesse contraindicação à ventilação prona ou essa fosse inefetiva em melhorar a PaO_2_/FiO_2_, manobra de recrutamento com níveis incrementais de PEEP (até 25cmH_2_O) seguida de titulação decremental da PEEP de acordo com a C_SR_ era realizada. Oxigenação por membrana extracorpórea e óxido nítrico inalado não estavam disponíveis nas UTIs participantes.

O modo ventilatório era modificado para pressão de suporte quando a PEEP estivesse ≤ 10cmH_2_O e os pacientes estivessem alertas o suficiente para respirar nessa modalidade ventilatória. O nível inicial de pressão de suporte era ajustado para alcançar um V_T_ de 6 a 8mL/kg PCP e uma FR de 12 a 30 incursões respiratórias por minuto (irpm). A pressão de suporte era reduzida em 2cmH_2_O a cada 2 a 4 horas, desde que o paciente mantivesse confortável e com V_T_ de 6 a 8mL/kg PCP e FR de 12 a 30irpm. O paciente era considerado apto para a extubação quando apresentasse estabilidade clínica, PaO_2_/FiO_2_ > 200mmHg, FR entre 8 e 30irpm e ausência de sinais de desconforto respiratório, por, pelo menos, 30 minutos de ventilação com pressão de suporte de 7cmH_2_O, PEEP ≤ 8cmH_2_O e FiO_2_ ≤ 0,4. Pacientes em VM por mais de 14 dias eram considerados para a realização de traqueostomia se apresentassem as seguintes indicações: expectativa de que a VM se prolongasse por, pelo menos, mais 7 dias, escore de Glasgow > 8 e deglutição ou reflexo de tosse inadequados com retenção de secreção. O desmame com traqueostomia seguia o mesmo protocolo.

### Desfechos

O desfecho primário foi a mortalidade hospitalar. Os desfechos secundários incluíam a mortalidade em 28 dias, mortalidade na UTI, duração da VM e tempo de permanência na UTI e no hospital.

### Análise estatística

O tamanho da amostra baseou-se no número de pacientes admitidos nas UTIs participantes e que respeitavam os critérios de inclusão/exclusão, sendo considerada uma amostra de conveniência. Assim, os achados deste estudo devem ser interpretados como exploratórios.

As variáveis contínuas foram expressas como mediana e intervalo interquartil (IQR) e empregou-se o Wilcoxon rank-test para a comparação; as variáveis categóricas foram expressas em número e percentual e comparadas pelo teste do qui-quadrado. As variáveis V_T_/kg PCP, P_plat_ e DP nos Dias 1 e 2 de VM foram apresentadas em gráficos de distribuição acumulada (*cumulative distribution plots*).

Modelos de regressão multivariados foram realizados para avaliar se V_T_/kg PCP e a DP estavam associados de forma independente com a mortalidade hospitalar. Em ambos os modelos, os fatores de confusão foram selecionados por meio de um gráfico acíclico direto (DAG - *direct acyclic graph*) (Figuras 1S e 2S - Material Suplementar). No modelo com V_T_/kg PCP como variável independente de interesse, os confundidores selecionados foram sexo, C_SR_, DP e FR. No modelo com a DP como variável independente de interesse, os confundidores selecionados foram C_SR_ e V_T_/kg PCP.

Também foi realizada análise de dupla estratificação para avaliar a associação entre DP e mortalidade hospitalar quando o V_T_/kg PCP era mantido constante. Inicialmente, foram criados seis quantis de V_T_/kg PCP. Depois, cada quantil foi estratificado em três quantis crescentes de DP. Finalmente, para cada estrato de V_T_/kg PCP foram fundidos os três *clusters* de DP, constituindo três subamostras com valores de V_T_/kg PCP semelhantes, porém com valores crescentes de DP. A análise de dupla estratificação foi repetida para obter três subamostras com valores semelhantes de DP, porém com valores crescentes de V_T_/kg PCP.

Todas as análises foram conduzidas com o Stata 15.1 (*Stata CorpLP, College Statio, TX, USA*), e o nível de significância foi de 0,05.

## RESULTADOS

Durante o período do estudo, 546 pacientes foram admitidos nas UTIs participantes, sendo que 296 preencheram os critérios de inclusão. Entre esses pacientes, 65 foram excluídos e 231 constituíram a coorte final. As principais razões para a exclusão foram: pacientes em VM invasiva por mais de 24 horas antes da admissão na UTI e pacientes que iniciaram cuidados paliativos pela equipe assistente (Figura 1).

**Figura 1:** Fluxograma dos participantes do estudo.

UTI - unidade de terapia intensiva.

A mediana de idade dos pacientes foi de 64 anos (IQR 53 - 74), 52,8% (122 pacientes) eram do sexo masculino, a mediana do SAPS 3 foi de 45 (IQR 39 - 54), e a SDRA foi classificada como moderada em 56,2% (121 pacientes) e grave em 19,5% (42 pacientes). As comorbidades mais prevalentes foram hipertensão arterial e diabetes (Tabela 1). A mortalidade hospitalar foi de 51,9% (120 pacientes). Os pacientes que sobreviveram eram mais jovens, tinham menor SAPS 3, menor SOFA e menor índice de comorbidade de Charlson. Diabetes, doenças cardiovasculares e doença renal crônica foram mais prevalentes entre os pacientes que não sobreviveram (Tabela 1). Pacientes que sobreviveram tinham menor DP e maior C_SR_, porém semelhantes V_T_/kg PCP, P_plat_, pressão de pico (P_peak_) e PEEP (Tabela 2 e Figura 2). A PaO_2_/FiO_2_ e o pH foram maiores nos pacientes que sobreviveram (Tabela 2).

**Tabela 1:** Características basais demográficas e clínicas dos pacientes

		Todos (n = 231)	Sobreviventes (n = 111)	Não sobreviventes (n = 120)	Valor de p
Idade (anos)		64 (53 - 74)	57 (46 - 65)	70 (60 - 78)	< 0,0001
Masculino		122 (52,8)	54 (48,6)	68 (56,6)	0,22
PCP (kg)		61,4 (52,4 - 67,8)	59,7 (52,4 - 66,0)	61,4 (52,4 - 70,5)	0,47
SAPS 3		45 (39 - 54)	41 (38 - 47)	51 (43 - 60)	< 0,0001
SOFA		3 (2 - 6)	3 (2 - 4)	4 (2 - 8)	0,0001
Índice de comorbidade de Charlson		3 (1 - 5)	2 (1 - 3)	4 (2 - 5)	< 0,0001
Comorbidades					
	Doença cardiovascular	36 (15,8)	11 (9,9)	25 (20,8)	0,02
	Doença renal crônica	17 (7,3)	4 (3,6)	13 (10,8)	0,03
	Doença pulmonar crônica	26 (11,2)	10 (9,0)	16 (3,3)	0,29
	Diabetes	91 (39,3)	33 (29,7)	58 (48,3)	0,004
	Hipertensão	152 (65,8)	66 (59,4)	86 (71,6)	0,051
	Obesidade	48 (20,7)	27 (24,3)	21 (17,5)	0,20
Gravidade da SDRA					0,10
	Leve	52 (24,1)	30 (28,5)	22 (20,0)	
	Moderada	121 (56,2)	60 (57,1)	61 (55,4)	
	Grave	42 (19,5)	15 (14,2)	27 (24,5)	

PCP - peso corporal predito; SAPS - *Simplified Acute Physiologic Score 3*; SOFA - *Sequential Organ Failure Assessment*; SDRA - síndrome do desconforto respiratório agudo. Resultados expressos como mediana (intervalo interquartil) ou n (%).

**Figura 2:** Parâmetros ventilatórios no primeiro dia de ventilação mecânica.

Distribuição de frequência acumulada da *driving pressure*, cmH_2_O (A) e volume corrente, mL/kg peso corporal predito (B). As linhas verticais representam os respectivos *cutoffs* de *driving pressure* e volume corrente para a ventilação mecânica protetora. V_T_ - volume corrente; PCP - peso corporal predito.

**Tabela 2:** Parâmetros respiratórios no Dia 1 de ventilação mecânica e desfechos clínicos

		Todos (n = 231)	Sobreviventes (n = 111)	Não sobreviventes (n = 120)	Valor de p
Modo de ventilação					0,15
	Pressão controlada	134 (58,0)	58 (52,2)	76 (63,3)	
	Volume controlado	96 (41,5)	52 (46,8)	44 (36,6)	
	Pressão de suporte	1 (0,4)	1 (0,90)	0	
Volume corrente (mL/kg de PCP)		6,48 (5,96 - 7,21)	6,54 (6,01 - 7,38)	6,43 (5,90 - 7,13)	0,17
Pressão de pico (cmH_2_O)		26 (24 - 29)	26 (24 - 29)	26 (24 - 30)	0,68
Pressão de platô (cmH_2_O)		24 (21 - 27)	24 (21 - 26)	24 (22 - 28)	0,39
*Driving pressure* (cmH_2_O)		13 (11 - 16)	12 (11 - 15)	14 (11 - 16)	0,03
PEEP (cmH_2_O)		10 (10 - 12)	10 (10 - 12)	10 (10 - 12)	0,63
C_SR_ (mL/cmH_2_O)		30.2 (24,6 - 36,6)	31,7 (26,2 - 36,6)	28,6 (23,3 - 35,0)	0,02
Ventilação prona*		134 (58,0)	60 (54,0)	74 (61,6)	0,24
FiO_2_		0,60 (0,50 - 0,75)	0,60 (0,45 - 0,70)	0,60 (0,50 - 0,80)	0,04
PaO_2_/FiO_2_ (mmHg)		195 (147,1 - 253,3)	210 (157,7 - 260)	187 (137,8 - 241,6)	0,06
PaCO_2_ (mmHg)		45 (40 - 53)	45 (40 - 53)	45 (39.4 - 52.9)	0,84
pH		7,34 (7,28 - 7,40)	7,35 (7,28 - 7,41)	7,33 (7,27 - 7,37)	0,01
Hemodiálise		63 (27,7)	17 (15,3)	46 (38,3)	< 0,0001
Duração da VM (dias)		13 (7 - 24)	10 (6 - 21)	15 (7 - 26)	0,07
Tempo de permanência na UTI (dias)		16 (9 - 28)	16 (10 - 28)	16 (7 - 29)	0,39
Tempo de permanência no hospital (dias)		22 (12 - 38)	27 (18 - 50)	16 (7 - 31.5)	< 0,0001
Duração da VM (dias)		13 (7 - 24)	10 (6 - 21)	15 (7 - 26)	0,07
Tempo de permanência na UTI (dias)		16 (9 - 28)	16 (10 - 28)	16 (7 - 29)	0,39
Tempo de permanência no hospital (dias)		22 (12 - 38)	27 (18 - 50)	16 (7 - 31,5)	< 0,0001

PCP - peso corporal predito; PEEP - pressão expiratória positiva final; C_SR_ - complacência do sistema respiratório; FiO_2_ - fração inspirada de oxigênio; PaO_2_/FiO_2_ - relação entre pressão parcial de oxigênio e fração inspirada de oxigênio; PaCO2 - pressão parcial de dióxido de carbono; VM - ventilação mecânica; UTI - unidade de terapia intensiva.

* Pacientes sob ventilação prona em qualquer momento durante a ventilação mecânica. Resultados expressos como n (%) ou mediana (intervalo interquartil).

Na análise multivariada, a DP se associou de forma independente com a mortalidade hospitalar. Por outro lado, não se encontrou associação estatística significante entre V_T_/kg PCP e mortalidade hospitalar (Tabela 3). A análise de dupla estratificação mostrou que, para um mesmo nível de V_T_/kg PCP, o risco de mortalidade hospitalar aumentava com a elevação da DP (Tabela 4). Entretanto, para o mesmo nível de DP, o risco de óbito no hospital não aumentou com o incremento do V_T_/kg PCP (Tabela 4).

**Tabela 3:** Regressão logística multivariável avaliando a associação entre volume corrente ou *driving pressure* e a mortalidade hospitalar

		Razão de chance (IC95%)	Valor de p
V_T_/kg PCP		0,75 (0,55 - 1,04)	0,09
	Sexo feminino	0,91 (0,45 - 1,85)	0,80
	C_SR_	1,03 (0,97 - 1,10)	0,30
	DP	1,20 (1,01 - 1,43)	0,03
	FR	1,01 (0,95 - 1,08)	0,62
DP		1,21 (1,04 - 1,41)	0,01
	C_SR_	1,03 (0,98 - 1,08)	0,15
	V_T_/kg PCP	0,74 (0,56 - 0,96)	0,02

IC95% - intervalo de confiança de 95%; V_T_ - volume corrente; PCP - peso corporal predito; C_SR_ - complacência do sistema respiratório; DP - *driving pressure*; FR - frequência respiratória. Associação entre volume corrente/kg de peso corporal predito e mortalidade hospitalar foi ajustada para sexo, complacência do sistema respiratório, *driving pressure* e frequência respiratória. Associação entre *driving pressure* e mortalidade hospitalar foi ajustada para complacência do sistema respiratório e volume corrente/kg de peso corporal predito.

**Tabela 4:** Análise de dupla estratificação: impacto do volume corrente/kg de peso corporal predito quando *driving pressure* é mantida constante e da *driving pressure* quando o volume corrente/kg de peso corporal predito é mantido constante

	Análise multivariável considerando o quantil 1 como referência (Três quantis de V_T_/kg de PCP crescente, com DP constante)		
	Quantil 1 (n = 76)	Quantil 2 (n = 77)	Quantil 3 (n = 78)
DP	13 (11 - 15)	14 (11 - 15)	13 (11 - 16)
V_T_/kg PCP	5,7 (5,4 - 6,0)	6,5 (6,3 - 6,8)	7,7 (7,2 - 8,5)
Mortalidade hospitalar (%)	57	58	41
Razão de chance (IC95%)		1,13 (0,59 - 2,20)	0,58 (0,30 - 1,12)
Valor de p		0,71	0,11
	Análise multivariável considerando o quantil 1 como referência (Três quantis de DP crescente, com V_T_/kg de PCP constante)		
	Quantil 1 (n = 94)	Quantil 2 (n = 71)	Quantil 3 (n = 66)
V_T_/kg PCP	6,5 (5,9 - 7,1)	6,6 (6,0 - 7,4)	6,4 (6,0 - 7,4)
DP	10 (9 - 12)	14 (13 - 15)	17 (16 - 18)
Mortalidade hospitalar (%)	44	56	59
Razão de chance (IC95%)		1,72 (0,92 - 3,25)	1,93 (1,02 - 3,71)
Valor de p		0,09	0,046

V_T_ - volume corrente; PCP - peso corporal predito; DP - *driving pressure*; IC95% - intervalo de confiança 95%. Análise multivariada ajustada para sexo e frequência respiratória.

Em relação aos desfechos secundários, a mortalidade em 28 dias foi de 37,2% (88 pacientes), e a mortalidade na UTI foi de 47,6% (110 pacientes). A mediana de permanência na UTI foi de 16 dias (IQR 9 - 28), e a mediana de permanência no hospital foi de 22 dias (IQR 12 - 38).

## DISCUSSÃO

Este estudo observacional avaliou uma coorte de pacientes com COVID-19 que preencheram os critérios de SDRA e foram submetidos à VM invasiva. Os resultados mostraram que a DP, que representa o V_T_ normalizado para a C_SR_, associou-se de forma independente com a mortalidade hospitalar. Por outro lado, quando o V_T_ era ajustado pelo PCP, não se encontrou associação estatisticamente significante com a mortalidade no hospital.

Desde 2000, quando o estudo ARMA mostrou que pacientes com SDRA ventilados com baixo V_T_ e baixa P_plat_ tinham mortalidade reduzida,^(17)^ diretrizes sobre o manejo da SDRA recomendam que esses pacientes recebam VM com limitação do V_T_ (4 - 8mL/kg PCP) e da P_plat_ (menos que 30cmH_2_O).^(9)^ Apesar dos benefícios de se ventilarem pacientes com SDRA com baixo V_T_, especialmente quando comparado com V_T_ de 12mL/kg PCP, ajustar o V_T_ apenas pelo PCP é criticado por alguns autores, porque não leva em consideração a gravidade da SDRA e a quantidade de tecido pulmonar que está disponível para a ventilação.^(16,18)^ Terragni et al., por exemplo, utilizando tomografia computadorizada para analisar pacientes com SDRA, demonstraram que, naqueles com grandes áreas de pulmão não aeradas (caracterizando o *small baby lung*), reduzir o V_T_ para 6mL/kg PCP e limitar a P_plat_ em 30cmH_2_O podem não ser suficientes para evitar a hiperinsuflação e minimizar a LPIV.^(16)^

Uma estratégia alternativa para reduzir o risco de LPIV em pacientes com SDRA seria ajustar o V_T_ para a C_SR_, ou seja, utilizar a DP (DP = V_T_/C_SR_) como parâmetro ventilatório de proteção. Como o estresse e o *strain* do tecido pulmonar, fatores envolvidos na fisiopatologia da LPIV, são consequência do V_T_ e do volume pulmonar ao fim da expiração, e esse último volume é determinado pela quantidade de tecido pulmonar aerado e representado pela C_SR_, a DP poderia ser considerada um melhor preditor da mortalidade do que o VT ou a P_plat_.^(19)^ A DP pode ser facilmente calculada a beira do leito, e diversos estudos observacionais mostraram sua associação com mortalidade.^(20,21)^ Além disso, Amato et al., analisando retrospectivamente dados de pacientes com SDRA incluídos em ensaios randomizados que compararam diferentes estratégias ventilatórias, mostraram que maiores níveis de DP foram preditores de menor sobrevida, enquanto níveis elevados de P_plat_ e V_T_ somente se associaram com menor sobrevida em pacientes com DP elevada.^(6)^

Nossos resultados, obtidos entre pacientes com COVID-19 que desenvolveram SDRA, estão em concordância com aqueles que mostraram que a DP é um melhor preditor de mortalidade do que o V_T_/kg PCP. Na nossa coorte, maiores níveis de DP, em oposição a maiores V_T_ baseados no PCP, associaram-se com maior mortalidade hospitalar. Além disso, em níveis constantes de V_T_/kg PCP, observamos que a DP se associou com a mortalidade, enquanto em níveis constantes de DP, o aumento do V_T_/kg PCP não se associou com maior risco de morte. Na verdade, na nossa coorte, encontramos um menor risco de óbito com o aumento do V_T_/kg PCP, apesar de não alcançar significância estatística. Esse achado pode refletir a estratégia de ajuste ventilatório adotada. Como baseamos nossa VM protetora em manter a DP abaixo de 15cm H_2_O, pacientes com menor C_SR_ podem ter sido ventilados com menor V_T_/kg PCP, resultando em associação entre baixo V_T_/kg PCP e maior mortalidade. No entanto, não podemos excluir que a redução do V_T_/kg PCP em pacientes com maior C_SR_ não é lesiva por si só, já que resulta no aumento do risco de assincronia e necessidades maiores de sedação e bloqueio neuromuscular.^(22,23)^ Esse impacto da mecânica respiratória no efeito da redução do V_T_ em pacientes com SDRA foi recentemente demonstrado. Os autores mostraram que o efeito da redução do V_T_ na mortalidade varia de acordo com a C_SR_ e que a diferença no risco de morte entre V_T_ alto e baixo somente foi significante estatisticamente em pacientes com baixa C_SR_.^(24)^

Nosso estudo tem várias limitações relevantes. A coorte foi constituída em apenas duas UTIs dedicadas a pacientes com COVID-19, que seguiam o mesmo protocolo de manejo ventilatório. Assim, os resultados não podem ser generalizados para outras UTIs. Os parâmetros ventilatórios foram coletados apenas nos 3 primeiros dias de VM e podem não representar aqueles aplicados nos dias seguintes, de tal forma que não podemos excluir a possibilidade de que os parâmetros ventilatórios e as terapias adjuvantes aplicados além do terceiro dia de VM influenciaram na mortalidade. Como é comum em estudos observacionais, o conhecimento da equipe da UTI a respeito do estudo que estava sendo conduzido poderia ter interferido na forma como os pacientes eram ventilados, aumentando a adesão à estratégia de VM protetora. Ao longo dos 15 meses de estudo, mudanças no manejo da COVID-19, não relacionadas à VM, bem como a experiência adquirida pelas equipes das UTIs, podem ter influenciado na sobrevida dos pacientes e o modelo causal não seria capaz de identificar esse aspecto. Nós não temos os dados a respeito do suporte ventilatório antes da intubação (cateter nasal de alto fluxo ou ventilação não invasiva). Assim, possíveis efeitos dessas estratégias não puderam ser avaliados no estudo. Como é um estudo observacional, as relações analisadas (coorte de COVID-19 com VM protetora e VM protetora com mortalidade hospitalar) podem ter sido influenciadas por confundidores residuais não incluídos nas DAGs. Portanto, a causalidade não pode ser assegurada.

## CONCLUSÃO

Na nossa coorte de pacientes com síndrome do desconforto respiratório agudo por COVID-19, a exposição a maior *driving pressure* se associou com maior mortalidade hospitalar. Contudo, não houve associação significativa entre o volume corrente ajustado pelo peso corporal predito com a mortalidade hospitalar. Esses resultados sugerem que a ventilação mecânica protetora poderia ter como alvo primário a *driving pressure* ao invés do volume corrente ajustado pelo peso corporal predito.
